# N-terminus of flagellin enhances vaccine efficacy against *Actinobacillus pleuropneumoniae*

**DOI:** 10.1186/s12917-022-03380-8

**Published:** 2022-07-16

**Authors:** Kamonpun Chuekwon, Chun-Yen Chu, Li-Ting Cheng

**Affiliations:** 1 Department of Tropical Agriculture and International Cooperation, International College, National Pingtung University of Science and Technology, 1, Shuefu Road, Neipu, Pingtung 91201 Taiwan; 2 Graduate Institute of Animal Vaccine Technology, College of Veterinary Medicine, National Pingtung University of Science and Technology, 1, Shuefu Road, Neipu, Pingtung 91201 Taiwan

**Keywords:** *Actinobacillus pleuropneumoniae*, Flagellin, Adjuvant, Pore-forming toxins

## Abstract

**Background:**

Flagellin elicits potent immune response and may serve as a vaccine adjuvant. We previously reported that the N-terminus of flagellin (residues 1–99, *n*FliC) is sufficient for vaccine efficacy enhancement against *Pasteurella multocida* challenge in chickens. In this study, we futher tested the adjuvancy of *n*FliC in a subunit vaccine against the pig pathogen *Actinobacillus pleuropneumoniae* in a mice model. For vaccine formulation, the antigen ApxIIPF (the pore-forming region of the exotoxin ApxII) was combined with *n*FliC, either through genetic fusion or simple admixture.

**Results:**

Immune analysis showed that *n*FliC, introduced through genetic fusion or admixture, enhanced both humoral (antibody levels) and cellular (T cell response and cytokine production) immunity. In a challenge test, *n*FliC increased vaccine protective efficacy to 60–80%, vs. 20% for the antigen-only group. Further analysis showed that, even without a supplemental adjuvant such as mineral salt or oil emulsion, genetically linked *n*FliC still provided significant immune enhancement.

**Conclusions:**

We conclude that *n*FliC is a versatile and potent adjuvant for vaccine formulation.

**Supplementary Information:**

The online version contains supplementary material available at 10.1186/s12917-022-03380-8.

## Background

Flagellin is the major structural protein of the bacterial flagellum. When recognized by Toll-like receptor 5 (TLR5) or NOD-like Receptor C4 (NLRC4), flagellin activates NF-κβ and initiates inflammatory immune responses that lead to adaptive immunity [1–3]. Therefore, flagellin has been investigated extensively as a vaccine adjuvant [4–7]. Structurally, flagellin from *Salmonella Typhimurium* can be divided into four domains: D0, D1, D2, and D3. In terms of protein sequence, flagellin domains are arranged as, starting at the N-terminus, D0-D1-D2-D3-D2-D1-D0. While D2 and D3 have been shown to be dispensable for TLR5 activation [8] D0 and D1 are highly conserved among bacteria species [8–10] and are essential for TLR5 signaling. Within D1, an important TLR5-binding hotspot is found at residues 89–96 [11–13]. The presence of D0 is absolutely required for TLR5 signaling and its role in TLR5 dimmerization has been suggested [14, 15].

In our previous study, we have attempted to further pinpoint the minimal region within D0 and D1 necessary for TLR5 activation and results indicated that the N-terminal portion of flagellin (residues 1–99, named *n*FliC) is sufficient for immune activation and vaccine efficacy enhancement [16]. *n*FliC contains parts of D0 and D1, including the important TLR5-binding hotspot, therefore it can be expect to retain significant TLR5 stimulatory activity. *n*FliC may be applied as an adjuvant to protein antigens.

*Actinobacillus pleuropneumoniae (A. pleuropneumoniae)* is one of the major bacterial pathogens affecting the swine industry. *A. pleuropneumoniae*, a Gram-negative bacterium, infects swine of all ages and causes respiratory diseases characterized by hemorrhagic, fibrinous and necrotic lung lesions. Inactivated vaccines are commercially available but provide limited heterologous protection [17]. For the development of subunit vaccines, the RTX (repeats-in-toxin) exotoxins ApxI, ApxII, and ApxIII have been shown to provide certain levels of protection as vaccine antigens [17, 18]. The 18 serotypes of *A. pleuropneumoniae* express different combinations of the Apx toxins [19]. ApxII is the most conserved among the serotypes, being expressed by all but sertoype 10 [17], making it a promising vaccine candidate [20]. We further identified the pore-forming region of ApxII (termed ApxIIPF) to be test as an antigen because of its lower molecular weight and importance in the hemolytic activity of the toxin [21, 22].

In this study, we evaluated the adjuvant effect of *n*FliC for a subunit vaccine against *A. pleuropneumoniae*. The antigen ApxIIPF was combined with *n*FliC, through either genetic fusion or simple admixture, and formulated as vaccines. Mice were immunized for immune response analysis and a challenge test was performed.

## Results

### *n*FliC was fused genetically or admixed with ApxIIPF as vaccines

**Fig. 1:**
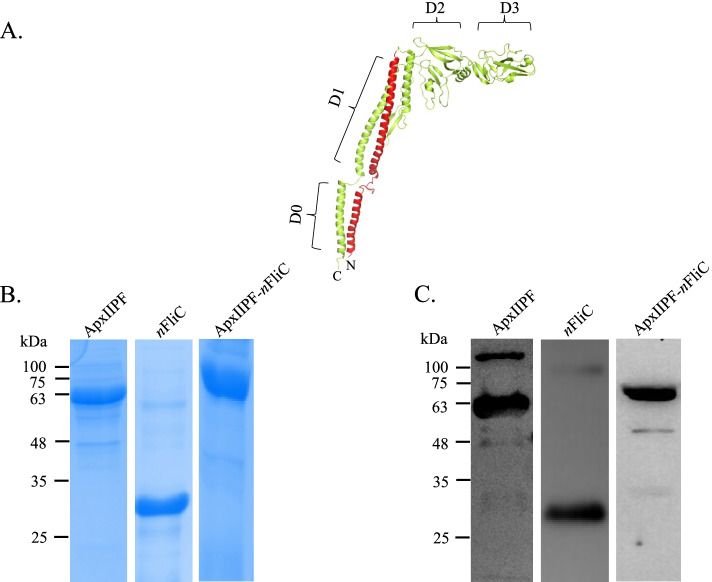
Construction and expression of the ApxIIPF-*n*FliC recombinant protein. **A** The 3-D structure of full-length flagellin (Protein Data Bank ID: 1UCU) as illustrated by the EzMol server is shown, with the N-terminus of flagellin, *n*FliC, labeled in red. **B** SDS-PAGE and and **C** Western blot analyses of the purified recombinant proteins are shown. Pictures of the SDS-PAGE gels and Western blot membranes are cropped for clarity and conciseness of presentation. See Supplementary Fig. 1 for original images

For vaccine formulation, *n*FliC (Fig. 1A) was combined as an adjuvant to the antigen, ApxIIPF, either through genetic fusion or in a simple admixture. ApxIIPF, *n*FliC, and the genetic fusion construct ApxIIPF-*n*FliC were expressed successfully in *Escherichia coli* (*E. coli*) at the expected molecular weights of 62, 30, and 73 kDa, respectively (Fig. 1B, note that the pET32a expression vector inserts a 20 kDa Trx-His-S-enterokinase tag). Identity of the recombinant proteins was reconfirmed with anti-His antibody in Western blot analysis (Fig. 1C). Using the recombinant proteins, five vaccines were formulated with or without the commercial water-in-oil-in-water adjuvant ISA 206 (since purifed protein antigens usually require additional supplemental adjuvants): (1) ApxIIPF+ISA 206 (2) ApxIIPF-*n*FliC + ISA 206 (3) ApxIIPF+*n*FliC + ISA 206 (4) ApxIIPF-*n*FliC and (5) PBS as the negative control. Mice were vaccinated twice for immune response analysis and challenge test.

### Genetically fused *n*FliC provided potent boost to antibody response

**Fig. 2:**
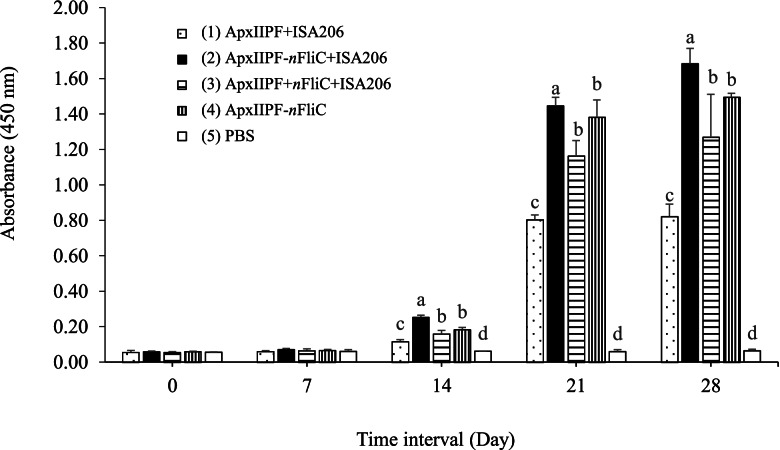
Antigen-specific antibody response of immunized mice. Mice (*n*=5) were immunized twice with the five vaccine formulations. Serum antibody levels were analyzed by indirect ELISA. Data are presented as mean ± SEM and different superscript letters indicate significant differences (*P* < 0.05) between treatment groups

The levels of antigen-specific antibody in immunized mice were measured using indirect ELISA with ApxIIPF as the coating antigen. Results show that ApxIIPF combined with the commercial adjuvant ISA 206 (vaccine Group 1) elicited antigen-specific antibody response (Day 21 and 28, Fig. 2), in contrast to the PBS group. When *n*FliC was added to the vaccine, either through genetic fusion (Group 2) or in a simple admixture (Group 3), antibody levels were significantly elevated (Day 21 and 28, *P* < 0.05) compared to the ApxIIPF+ISA 206 group, demonstrating an adjuvant effect. Furthermore, the enhancement effect was more pronounced for the genetic fusion group (Group 2) than the admixture group (Group 3). Interestingly, even without the commercial adjuvant (Group 4), the ApxIIPF-*n*FliC construct elicited a higher antibody level than Group 1 vaccine, obviating the need for ISA 206. Overall, results demonstrate that genetically linked *n*FliC boosted antibody production, even in the absence of a supplemental adjuvant.

### *n*FliC enhanced CD4^+^ and CD8^+^ T cell expansion

**Fig. 3:**
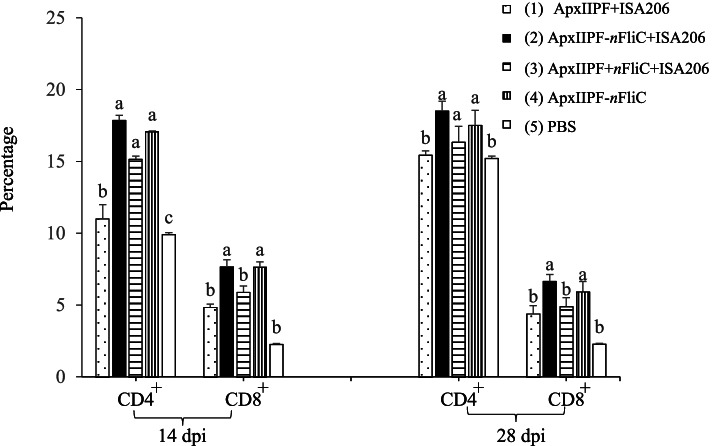
Percentage of CD4^+^ and CD8^+^ T cells in the splenocytes of immunized mice. Mice (*n*=2) were immunized twice with the five vaccine formulations and isolated splenocytes were stained with anti-CD4^+^ and CD8^+^ antibody for flow cytometry analysis. Data are presented as mean ± SEM and different superscript letters indicate significant differences (*P* < 0.05) between treatment groups at the same time point for CD4^+^ and CD8^+^ T cells

To determine the extent of T cell stimulation by the vaccines, the percentages of CD4^+^ and CD8^+^ T cells in the splenocytes were analyzed for immunized mice. On Day 14, CD4^+^ T cell percentages of *n*FliC-containing vaccine groups (Group 2, 3, and 4) were all significantly higher (*P* < 0.05) than the non-*n*FliC-containing Group 1 (Fig. 3). Similar enhancement was observed for CD8^+^ T cells except for the admixture group (Group 3). These results indicate that *n*FliC augmented cellular immune response. The difference between the groups became less prominent by Day 28.

### Genetically fused *n*FliC increased gene expression of proinflammatory, T_H_1, and T_H_2-type cytokines

**Fig. 4:**
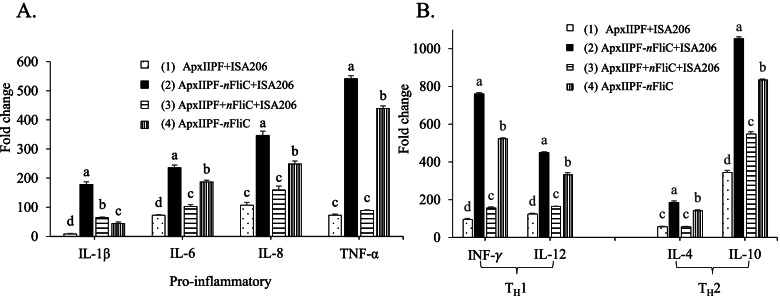
Cytokine gene expression of splenocytes from immunized mice. Mice (*n*=2) were immunized twice with the five vaccine formulations and isolated splenocytes from Day 28 were stimulated with purified antigens. Relative mRNA expression levels of various cytokines were determined, (**A**) pro-inflamatory cytokines (**B**) T helper cells. Data are presented as mean ± SEM and different superscript letters indicate significant differences (*P* < 0.05) between treatment groups

To characterize the type of immune response elicited by the vaccines, cytokine expression profile of splenocytes from immunized mice (Day 28) were determined. Results showed that *n*FliC significantly enhanced cytokine expression (IL-1β, IL-6, IL-8, TNF-α, IFN-γ, IL-12, IL-4, and IL-10, *P* < 0.05) for the *n*FliC-containing vaccine groups (Group 2, 3, and 4), when compared to the non-*n*FliC-containing Group 1 (Fig. 4A and B). Overall, genetically fused *n*FliC provided the most enhancement in cytokine expression while the admixed *n*FliC provided relatively lower amount of boost.

### Genetically fused *n*FliC enhanced vaccine protection rate in a challenge test

**Fig. 5:**
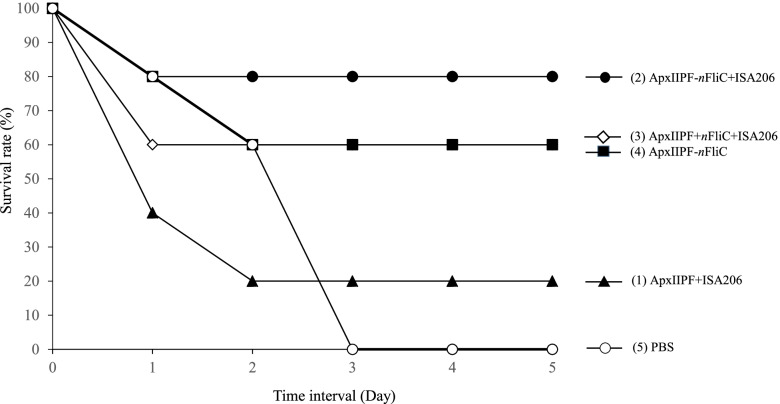
Survival rate of immunized mice when challenged with *A. pleuropneumoniae*. Mice (*n* = 5) were immunized twice the five vaccine formulations and challenged with 10 LD_50_ (5 × 10^7^ CFU/dose) *A. pleuropneumoniae* strain 4707

**Fig. 6:**
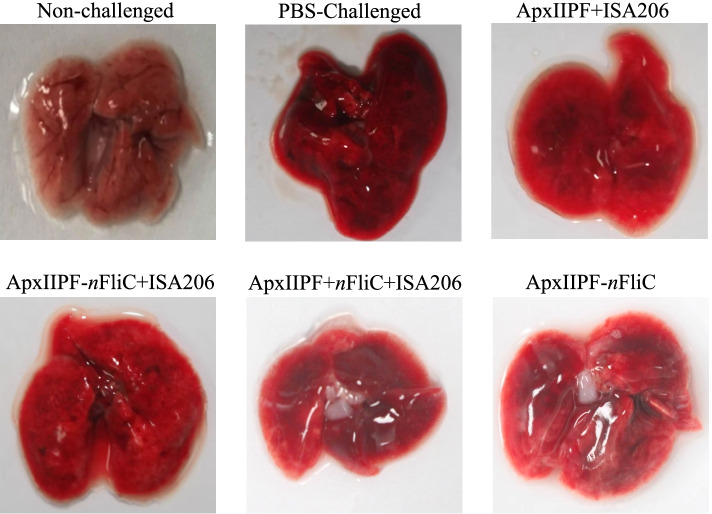
Gross lesion of the lungs after challenge with virulent *A. pleuropneumoniae* strain 4707. Mice (*n* = 5) were immunized twice the five vaccine formulations and challenged with 10 LD_50_ (5 × 10^7^ CFU/dose) *A. pleuropneumoniae* strain 4707. Examples of lungs of sacrificed mice are shown

Vaccinated mice were challenged with *A. pleuropneumoniae*. The survival rate for the ApxIIPF+ISA206 vaccine group was 20% (*n* = 5). In contrast, the *n*FliC-containing vaccine groups showed 60–80% protection rates, with the ApxIIPF-*n*FliC + ISA206 group being the highest (Fig. 5). This demonstrates that *n*FliC provided a significant boost to protective efficacy. Examples of lung gross lesions of sacrificed mice are shown in Fig. 6, with the vaccine groups showing significantly less bleeding than the challenged PBS group.

## Discussion

As a continuation study to our previous work with *Pasteurella multocida* in chickens [16], this work further demonstrated the adjuvant effect of *n*FliC using a pig pathogen, *A. pleuropneumoniae*. Elevated immune reponse and enhanced protective efficacy were observed when *n*FliC was included in the vaccine through genetic fusion or simple admixture. Closer examination of the immune response and protective efficacy results indicated that the genetic fusion method provided superior immune boost compared to the admixture method. Furthermore, the genetic fusion method afforded sufficient immune enhancement such that additional adjuvants may be optional.

Literature showed that when using flagellin as an adjuvant (the full length or the D0/D1 domains), the genetic fusion method elicits faster and more potent immune response than the admixture method [23–26]. In our study, the N-terminus of flagellin also showed superior immune enhancement when it is genetically linked to the antigen versus through an admixture. However, we do note that, in the presence of the supplemental adjuvant ISA 206, the admixture vaccine elicited immune enhancement levels (antibody levels and protection rate) not so drastically lower than that of the genetic fusion vaccine (Group 2 vs. 3), making the admixture method a feasible option in vaccine formulation. Simple admixture of antigen and adjuvant may be a desirable option in terms of vaccine design, industrial scale production, and vaccine portfolio strategy.

Pure proteins used as vaccine antigens are usually not immunogenic and thus require the presence of supplmental adjuvants such as mineral salts or oil emulsions for fast and potent immune response [27]. In our study, we sought to determine if immune stimulation by *n*FliC would obviate the need for additional adjuvants. Results showed comparable levels of humoral and cellular immune response for the ApxIIPF-*n*FliC construct with or without the ISA 206 adjuvant (Group 2 vs. 4). Protective efficacy of the ApxIIPF-*n*FliC construct was 80 and 60% with and without ISA 206, respectively. Therefore, additional boost provided by ISA 206 appears limited and may be considered optional in the presence of a genetically linked *n*FliC.

When the immunostimulatory profiles of *n*FliC in two different disease models (*A. pleuropneumoniae* in this study and *P. multocida* in chickens in our previous study [16]) are compared, we find them to remain fairly consistent. Antibody production enhancement and cellular immune response profiles were similar for both disease studies. Furthermore, analysis of cytokine expression profiles of our two studies, along with others [28], demonstrated that flagellin elicits a more balanced T_H_1 and T_H_2 immune response. It is promising that the adjuvant effect of flagellin can be applied consistently to antigens of various pathogens.

## Conclusions

In summary, we find that the genetic fusion method of combining *n*FliC with the antigen ApxIIPF provides superior immune enhancement over the admixture method. Furthermore, genetically linked *n*FliC obviates the need for supplemental adjuvants. However, if the admixture method is desired for vaccine formulation purposes, additional supplemental adjuvants would be required.

## Methods

### Bacteria strains

*A. pleuropneumoniae* strain 4707 (serotype 1, ATCC® 27088™) obtained from the American Type Culture Collection (ATCC) was cultured in Brain-Heart Infusion broth supplemented with 15 mg/mL nicotinamide adenine dinucleotide (NAD) at 37 °C.

### Cloning and protein expression of ApxIIPF, *n*FliC, and ApxIIPF-*n*FliC

ApxIIPF, defined as the first 380 residues of ApxII [29], was cloned from the genomic DNA of *A. pleuropneumoniae* strain 4074 by PCR using a primer set listed in Table 1. PCR product was digested with BamH I and Xho I, and the amplicon was ligated into the expression vector pET32a (Novagen, Darmstadt, Germany). *E. coli* DH5α (Yeastern Biotech, Taipei, Taiwan) was used for plasmid propagation and the construct was send for sequence confirmation. *n*FliC was cloned in a similar fashsion from the genomic DNA of *S. typhimurium* (ATCC® 14028™) using PCR primers (Table 1), as reported previously [16]. To construct the ApxIIPF-*n*FliC fusion gene, chimeric PCR was perfomed using *n*FliC and ApxIIPF as templates and primers for ApxIIPF-*n*FliC (Table 1). Note that *n*FliC was linked to the N-terminus of ApxIIPF through a glycine-serine linker. The construct was inserted into pET32a and the sequence reconfirmed.

**Table 1:** Primers for gene cloning and recombinant protein construction

Target gene	Oligonucleotide Sequence (5′ to 3′)	RE site	Gene length (bp)	NCBI Reference Sequence
*n*FliC	F: *GGATCC*ATGGCACAAGTCATTAATACAAAC	*Bam*HI	312	ATCC® 14,028™
	R: GCGCTCGAGAGACTGAACCGCCAGTTC	*Xho*I		
ApxIIPF	F: GC*GGATCC*ATGTCAAAAATCACTTTGTCATCAT	*Bam*HI	1140	ATCC® 27088™
	R: CGCTCGAGAGCTCCAACTCCACCGGAGAT	*Xho*I		
*n*FliC-gs	F: *GGATCC*ATGGCACAAGTCATTAATACAAAC	*Bam*HI-	312	*n*FliC
	R: **GCTGCCGCCCCCGCC**AGACTGAACCGCCAGTTC			
ApxIIPF-gs	F: **GGCGGGGGCGGCAGC**ATGTCAAAAATCACTTTGTCATCAT	-*Xho*I	1140	ApxIIPF
	R: CGCTCGAGAGCTCCAACTCCACCGGAGAT			
ApxIIPF-*n*FliC	F: *n*FliC F	*Bam*HI *Xho*I	1467	ApxIIPF and *n*FliC
	R: ApxIIPF R			

*Bam*HI site: Italic; *Xho*I site: Underlined; glycine-serine linkers: Bold fonts

To express the recombinant proteins, as described previously [16], plasmid constructs were used to transform chemically competent *E. coli* BL21(DE3) (Yeastern Biotech, Taipei, Taiwan) according to the manufacturer’s instructions. Protein expression was induced by adding 1 mM isopropyl-β-D-thiogalactopyranoside (IPTG; Sigma, Darmstadt, Germany) for 4 hours at 37 °C. Cells were harvested, lysed in native lysis buffer (300-mM KCl, 50-mM KH_2_PO_4_ and 5-mM Imidazole) and sonicated. The soluble fraction was used for recombinant protein purification through the His-tag with Bio-scale Mini Profinity IMAC cartridges (1 mL) (Bio-Rad, Hercules, CA, USA) according to the manufacture’s instructions. The quality and quantity of the recombinant protein were analyzed using 12% sodium dodecylsulfate-polyacrylamide gel electrophoresis (SDS-PAGE). Western blotting was also performed to confirm the identity of the expressed proteins. 6X-His Tag Antibody (Gentex, Hsinchu, Taiwan) at 1:5000 dilution was used as primary antibody, and peroxidase-conjugated goat anti-mouse antibody (Gentex, Hsinchu, Taiwan) at 1:5000 dilution was used as secondary antibody. Western Lighting PLUS (PerkinElmer, Waltham, MA, USA) was used for color development. Using the ToxinSensor™ Chromogenic LAL Endotoxin AssayKit (GenScript, Piscataway, NJ, USA), endotoxin levels of the purified proteins were confirmed to be less than 0.125 EU/mL.

### Vaccine formulation and immunization

To evaluate the adjuvant effect of *n*FliC, five vaccines were formulated with the recombinant proteins: (1) ApxIIPF+ISA 206 (2) ApxIIPF-*n*FliC + ISA 206 (3) ApxIIPF+*n*FliC + ISA 206 (4) ApxIIPF-*n*FliC and (5) phosphate-buffered saline (PBS) as the negative control group. Purified recombinant proteins (at 25 μg/dose final concentration) were formulated with or without the commercial water-in-oil-in-water emulsion adjuvant MONTANIDE™ ISA 206 (Seppic, Courbevoie, France) in a 1:1 ratio. A final volume of 200 μL/dose is used for vaccination of mice.

For immmunization, a total of 45 five-week-old female ICR mice from BioLASCO Taiwan Co., Ltd., (Taipei, Taiwan) were randomly divided into five groups of nine mice each, for the five vaccine formulations. Mice were immunized twice intramuscularly on Day 0 and 14. Blood samples were collected weekly after primary immunization for immune response analysis. On Day 14 and 28, splenocytes from two mice from each vaccine group were isolated for T-cell response analysis. All animal experimental protocols (NPUST-109-064) were approved by the Insititutional Animal Care and Use Committee of the National Pingtung University of Science and Technology, Taiwan.

### Analysis of humoral immune response

Serum titers of antigen-specific IgG were determined via indirect ELISA. Collected whole blood was permitted to coagulate and then centrifuged at 500×*g* for 7 min to obtain serum. ELISA plates were coated overnight with 5 μg/mL of ApxIIPF in 50 mM carbonate buffer (pH 9.6) at 4 °C and then blocked for 1 h at 37 °C with a blocking buffer (5% skim milk in PBS). Plates were then washed with PBST (PBS with 0.1% Tween 20). Serum samples were serially diluted at a 1:5 ratio seven times (total dilution of 1:78,125) for incubation as the primary antibody for 1.5 h at 37 °C. The plates were washed with PBST before the addition of the secondary antibody, Anti-Mouse IgG Antibody (HRP) (GeneTex, Irvine, CA, USA) at 1:5000 dilution. Finally, the Peroxidase Kit (KPL, Gaithersburg, MD, USA) was used for color development and the plates were read at 450 nm on the Multiskan™ FC Photometer (Thermo Fisher Scientific, Vantaa, Finland).

### Analysis of cellular immune response

To isolate splenocytes for T-cell response analysis, two mice from each vaccine group were sacrificed by cervical dislocation method on Day 14 and 28. Harvested spleens were mashed through cell strainers and spleen cells were collected in RPMI 1640 Media (Gibco Invitrogen, Carlsbad, CA, USA). Erythrocytes were lysed with ACK lysis buffer (150 mM NH_4_Cl, 10 mM KHCO_3_, 0.1 mM Na_2_-EDTA, pH 7.4) and the remaining splenocytes were wash three times with PBS before resuspension (at 1 × 10^6^ cells/mL) in RPMI-1640 Media supplemented with 5% fetal bovine serum (Gibco Invitrogen, Carlsbad, CA, USA).

To analyze the percentage of CD4^+^ and CD8^+^ T-cell populations of the splenocytes, prepared cells (1 × 10^6^ cells/mL) were resuspended in PBS and incubated for 30 min at 4 °C with the fluorescently labeled anti-CD4-APC, anti-CD8-FITC antibodies according to the manufacturer’s technical bulletin (Sino Biological Inc., Wayne, PA, USA). Then, preparations were washed three times with PBS. Labeled cells were analyzed using the BD Accuri™ C6 flow cytometer (BD Biosciences, San Diego, CA, USA).

To determine the cytokine response of the splenocytes from vaccinated mice (Day 28), cells (1 × 10^6^ cells/mL) were stimulated with 10 μg/mL of ApxIIPF for 3 hours. Total RNA was then extracted with the Total RNA Extraction Miniprep system (Viogene, Taipei, Taiwan) and complementary DNA was synthesized using the Reverse Transcription Kit (Applied Biosystems, Foster, CA, USA). Real-time PCR was then carried out in the SmartCycler I (Cepheid, Sunnyvale, CA, USA) with primers for the cytokines listed in Table 2. Expression levels of the cytokine genes were normalized to that of the housekeeping gene glyceraldehyde-3-phosphate dehydrogenase (GAPDH). Proinflammatory (IL-1β, IL-6, IL-8, and TNF-α), T_H_1-type (IFN-γ and IL-12), and T_H_2-type (IL-4 and IL-6) cytokine levels were determined by real-time PCR and expressed as N-fold increase or decrease relative to that of the PBS group [30].

**Table 2:** Primers for cytokine genes

Gene	Oligonucleotide Sequence (5′ to 3′)	Tm (°C)	Gene length (bp)	NCBI Reference Sequence
IL-1β	F:AGTTGACGGACCCCAAAAGAT	57	412	M15131.1
	R: CATGGAGAATATCACTTGTT			
IL-4	F:CGAAGAACACCACAGAGAGTGAGCT	50	175	M25892.1
	R:GACTCATTCATGGTGCAGCTTATCG			
IL-6	F:CTTCCATCCAGTTGCCTTCTTG	57	141	M24221.1
	R:AATTAAGCCTCCGACTTGTGA			
IL-8	F: CAAGGGCCAAGAGAATATCC	55	445	BC013615.1
	R: TTACTATAACATCTTTATAA			
IL-10	F: AAGGCAGTGGAGCAGGTGAA	55	155	NM_010548.2
	R: CCAGCAGACTCAATACACAC			
IL-12p40	F: CAGAAGCTAACCATCTCCTGGTTTG	55	396	BC103610.1
	R: CCGGAGTAATTTGGTGCTCCACAC			
IFN-γ	F:AGCGGCTGACTGAACTCAGATTGTAG	55	243	NM_008337.4
	R:GTCACAGTTTCAGCTGTATAGGG			
TNF-α	F:GGCAGGTCTACTTTGGAGTCATTGC	55	300	NM_001278601.1
	R:ACATTCGAGGCTCCAGTGAATTCGG			
GADPH	F: CGGCACAGTCAAGGCCGAGAAT	57	154	M32599.1
	R:AGCCTTCTCCATGGTGGTGAA			

*Note*: *IL* Interleukin, *IFN-γ* Interferon gamma, *TNF-α* Tumor Necrosis Factor alpha, *GAPDH* Glyceraldehyde 3 phosphate dehydrogenase

### *A. pleuropneumoniae* challenge test

On Day 28 after primary immunization, vaccinated mice (*n* = 5) were challenged intramuscularly with 5 × 10^7^ CFU (10 LD_50_) of *A. pleuropneumoniae* 4707. Mice were observed for clinical signs and survival rates recorded. Mice were monitored for 5 days and moribund mice satisfying criteria for humane endpoints (as defined by the Animal Use Protocol of NPUST) were sacrificed by cervical dislocation. Post-mortem dissections were performed to obtain the lungs for observation. All mice were sacrificed at the end of the five-day period.

### Statistical analysis

One-way analysis of variance (ANOVA) and Student’s *t*-test were used for comparison between groups. Data were expressed as mean ± standard error of mean (SEM) and *P* value less than 0.05 was considered statistically significant. All statistical analyses were performed using SAS version 9.0.

## Supplementary Information

**Additional file 1: Supplementary Fig. 1.** Original files of SDS-PAGE and Western blots of purified recombinant proteins. SDS-PAGE and Western blot analysis were performed to verify recombinant protein production and identity.

## Data Availability

The datasets supporting the conclusions of this article are included within the article.

## Abbreviations

ApxIIPF: Pore-forming domain of ApxII from *A. pleuropneumoniae*
*n*FliC: N-terminal of flagellin from *S. typhimurium*
ApxIIPF-*n*FliC: N-terminal of flagellin fused to pore-forming domains of ApxII
