# Supplementary material for: N-terminus of flagellin enhances vaccine efficacy against Actinobacillus pleuropneumoniae
Source: BMC Vet Res. 2022 Jul 16;18:279. doi: 10.1186/s12917-022-03380-8 (PMC9288005; doi:10.1186/s12917-022-03380-8)
Supplement: Supplementary file 1 — Additional file 1: Supplementary Fig. 1. Original files of SDS-PAGE and Western blots of purified recombinant proteins. SDS-PAGE and Western blot analysis were performed to verify recombinant protein production and identity. [file 12917_2022_3380_MOESM1_ESM.pptx]

## Slide 1
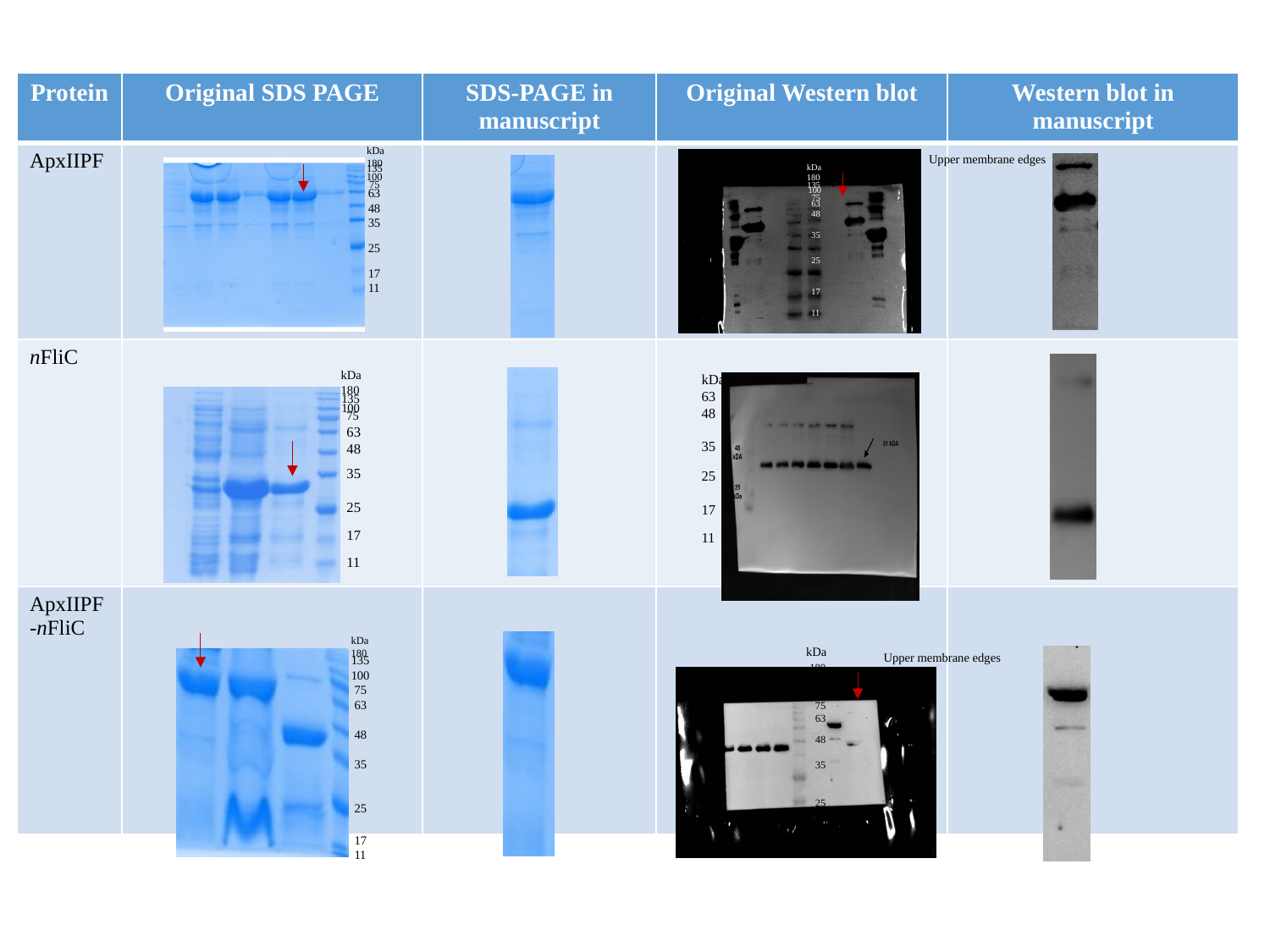

| Protein | Original SDS PAGE | SDS-PAGE in manuscript | Original Western blot | Western blot in manuscript |
| --- | --- | --- | --- | --- |
| ApxIIPF | | | | |
| nFliC | | | | |
| ApxIIPF-nFliC | | | | |
kDa
180
63
48
35
25
17
11
100
Upper membrane edges
kDa
180
135
135
75
100
100
75
63
48
35
25
17
11
63
48
35
25
17
11
kDa
180
75
63
48
35
25
17
11
kDa
63
48
35
25
17
11
135
100
kDa
180
kDa
 180
 135
 100
 75
 63
 48
 35
 25
 17
 11
Upper membrane edges
135
100
 75
 63
 48
 35
 25
 17
 11

## Slide 2
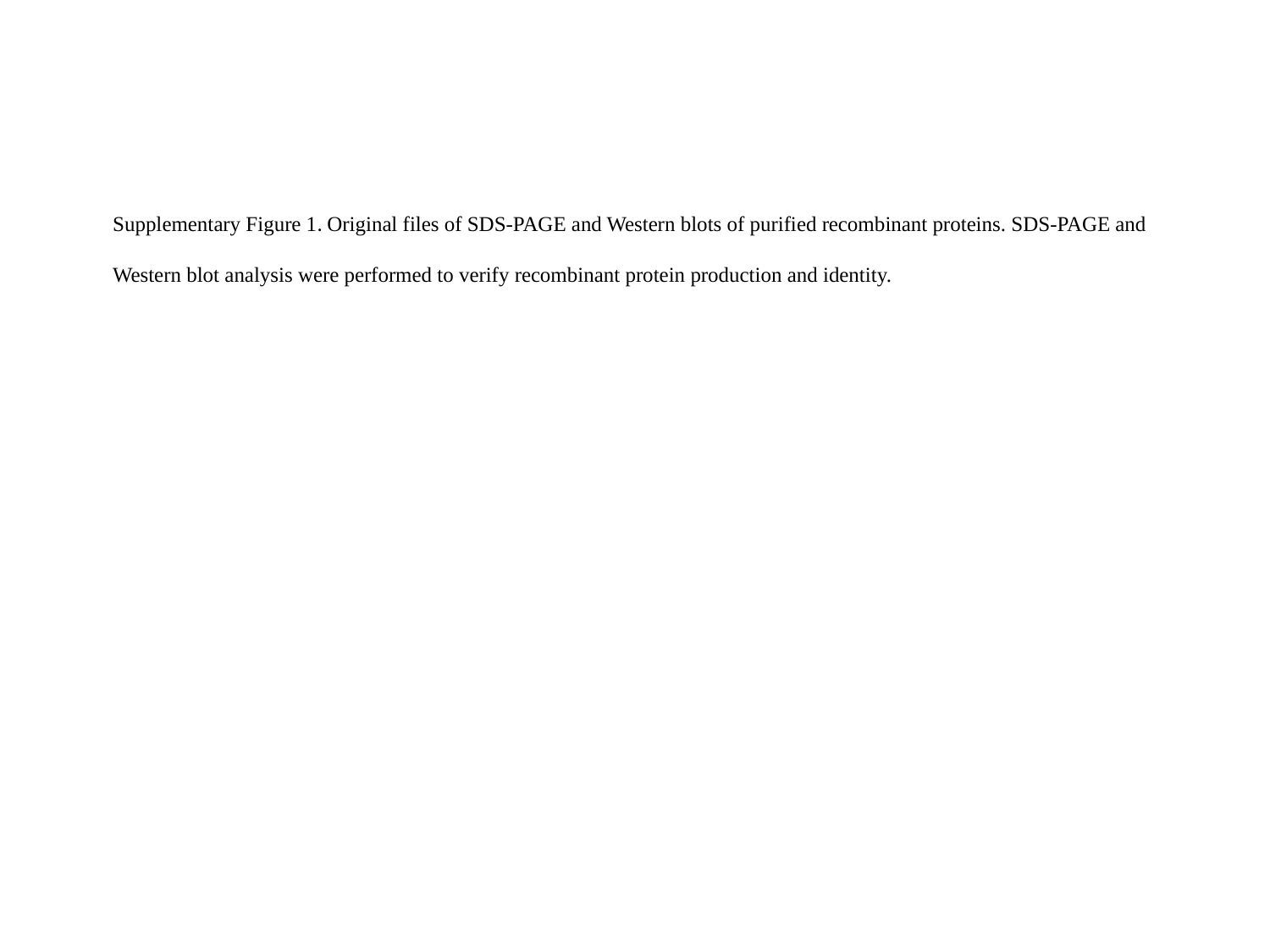

Supplementary Figure 1. Original files of SDS-PAGE and Western blots of purified recombinant proteins. SDS-PAGE and Western blot analysis were performed to verify recombinant protein production and identity.
